# Between-domain relations of students' academic emotions and their judgments of school domain similarity

**DOI:** 10.3389/fpsyg.2014.01153

**Published:** 2014-10-21

**Authors:** Thomas Goetz, Ludwig Haag, Anastasiya A. Lipnevich, Melanie M. Keller, Anne C. Frenzel, Antonie P. M. Collier

**Affiliations:** ^1^Department of Empirical Educational Research, University of KonstanzKonstanz, Germany; ^2^Department of Empirical Educational Research, Thurgau University of Teacher EducationKreuzlingen, Switzerland; ^3^Department of Educational Psychology, University of BayreuthBayreuth, Germany; ^4^Department of Educational Psychology, Queens College and the Graduate Center, The City University of New YorkNew York, NY, USA; ^5^Department of Psychology, University of MunichMunich, Germany

**Keywords:** domain-specificity, academic domains, emotions, trait, state

## Abstract

With the aim to deepen our understanding of the between-domain relations of academic emotions, a series of three studies was conducted. We theorized that between-domain relations of trait (i.e., habitual) emotions reflected students' judgments of domain similarities, whereas between-domain relations of state (i.e., momentary) emotions did not. This supposition was based on the accessibility model of emotional self-report, according to which individuals' beliefs tend to strongly impact trait, but not state emotions. The aim of Study 1 (interviews; *N* = 40; 8th and 11th graders) was to gather salient characteristics of academic domains from students' perspective. In Study 2 (*N* = 1709; 8th and 11th graders) the 13 characteristics identified in Study 1 were assessed along with academic emotions in four different domains (mathematics, physics, German, and English) using a questionnaire-based trait assessment. With respect to the same domains, state emotions were assessed in Study 3 (*N* = 121; 8th and 11th graders) by employing an experience sampling approach. In line with our initial assumptions, between-domain relations of trait but not state academic emotions reflected between-domain relations of domain characteristics. Implications for research and practice are discussed.

## Introduction

Empirical educational research has largely neglected the role of students' emotional experiences, with the exception of extensive research on test anxiety (Sarason and Mandler, 1952; Zeidner, 1998, 2007) and on emotions in achievement settings based on attribution theory (see Weiner, 1985, 2001). However, over the past decade, as a consequence of recognizing the importance of academic emotions, defined as “emotions that are directly linked to academic learning, classroom instruction, and achievement” (Pekrun et al., 2002, p. 92), theoretical and empirical contributions increased significantly in this field. This is reflected in a number of recent special issues in flagship journals and edited volumes devoted to the subject (Schutz and Lanehart, 2002; Efklides and Volet, 2005; Linnenbrink, 2006; Schutz and Pekrun, 2007; Linnenbrink-Garcia and Pekrun, 2011; Lipnevich and Roberts, 2012; Pekrun and Linnenbrink-Garcia, 2014). A significant number of studies has focused on the antecedents, effects, and structure of academic emotions (e.g., research on the structure of enjoyment; Goetz et al., 2006a). An important area of research related to the latter topic is the investigation of between-domain relations of academic emotions (e.g., Goetz et al., 2007), with studies examining the degree to which students' emotional experiences in one academic domain (e.g., mathematics) are related to their experiences in another domain (e.g., English). This topic is important, as understanding the nature of between-domain relations of academic emotions can guide assessment (i.e., domain-specific vs. domain-general measurement) and can inform teachers of whether or not making generalizations about students' emotions from one domain to another are warranted.

Existing research indicates that between-domain relations of academic emotions are, on average, relatively weak, providing evidence that academic emotions are organized largely in a domain specific way (e.g., Stipek and Mason, 1987; Marsh and Yeung, 1996; Goetz et al., 2006b, 2007, 2012b). Further, the revealed relations between emotions experienced in two different domains vary in strength, with some domain pairs, such as mathematics and physics, showing stronger relations, and other domains being virtually unrelated (e.g., mathematics and English, Goetz et al., 2007). It is important to note that almost all research on this topic is based on assessing trait (habitual) emotions (e.g., Stipek and Mason, 1987; Marsh and Yeung, 1996).

Beyond empirical evidence showing that academic emotions are organized in a domain-specific way, knowledge is lacking on *why* they are domain-specific in nature and, more specifically, why the relations between emotions associated with some domain pairs (e.g., mathematics/physics) are stronger than those associated with others (e.g., mathematics/English). Goetz et al. (2011) stated that various academic domains may be perceived as more or less similar, and the degree of similarity may explain why relations between emotions experienced in those domains may be more or less strong. In other words, students who view certain domains as similar may be expected to also report similar emotions related to those domains.

The latter contingency, however, primarily applies to trait emotions that are known to be strongly impacted by individuals' belief systems (Robinson and Clore, 2002; Goetz et al., 2013; Bieg et al., 2014). So, cognitions related to characteristics of domains become critical when students reflect on their habitual emotions related to those domains. In contrast, such cognitions are substantially less likely to have an impact on real-time academic emotions as experienced by students in a given situation, referred to as state emotions. When reporting state emotions, students refer to “here and now” and cognitions appear to play a subordinate role in such reports. For example, students may judge mathematics and physics as being more similar in their characteristics as compared to mathematics and English, and therefore students' academic emotions (e.g., enjoyment, anxiety) in mathematics may be more strongly related to their emotions in physics as compared to English. Given, however, that domain judgments may have a stronger impact on the report of habitual (trait) emotions, trait assessment should be more sensitive to similarities or dissimilarities in domain pairings than students' report of real-time academic emotions. The focus of the present research was to investigate whether between-domain relations of trait academic emotions reflected cognitions about subject domains. We hypothesized that the degree of similarity in the relational pattern of academic emotions on the one hand, and students' judgments of between-domain characteristics on the other, depended upon the method of assessment. That is, the degree of similarity was expected to be stronger for the trait as compared to the state assessment of academic emotions.

## Judgments of domain characteristics and between-domain relations of academic emotions

At first glance, some academic domains are intuitively judged as being more similar than others. However, when thinking about *why* two specific domains should in fact be more similar than other domain pairs, some questions arise: How can we compare any two domains? What facets or dimensions of domains are most salient for such comparisons? And finally: What characteristics describe a domain? A rather straightforward approach to determining whether or not two academic domains are similar is to compare these domains on a number of selected characteristics. One might argue, for example, that mathematics and physics are similar with respect to content difficulty or that mathematics and English are rather different with respect to the coverage of topics that are currently discussed in society. Judgments of domains may differ depending on the person making them. For example, they may vary from students to parents and teachers or to developers of curricula and researchers. Because the goal of the present research was to relate student judgments of domains to the between-domain relations of their academic emotions, we exclusively focused on *student* judgments about domain characteristics.

Studies looking at students' perspectives on academic domains mainly appear in the area of didactics. They typically focus on *one* specific domain and its characteristics (mainly mathematics, e.g., Cobb et al., 1991; Carpenter et al., 1999). Characteristics outlined in these studies tend to be “top-down,” that is, based on the work of professionals in those domains (e.g., Schreiner and Sjøberg, 2004, for science education). It is implicitly assumed that these characteristics are salient for students as well, with no studies available to date to support this assumption.

Further, the issue of domain-salient features for students also arises when examining studies rooted in educational psychology, where domain characteristics are typically gleaned from teachers and university professors (e.g., Biglan, 1973; Donald, 1983, 1990, 1995; Stodolsky, 1993; Becher, 1994; Stodolsky and Grossman, 1995, 2000). In this line of research “domain knowledge” is the umbrella term that has been used in a number of studies to describe various characteristics of domains (see Alexander, 1992, for an overview). It can be defined as “the realm of knowledge that individuals have about a particular field of study” (Alexander, 1992, p. 34; see also Alexander and Judy, 1988; Alexander et al., 2012). Although researchers working within the domain knowledge perspective have looked into what comprises academic domains, comparing a number of domains with respect to specific facets appears to be an auxiliary meta-level issue within this research.

Yet another area of research that describes beliefs that students hold about different academic domains focuses specifically on students' epistemological beliefs (i.e., beliefs about the nature of knowledge and the nature of knowing; Perry, 1970; Hofer and Pintrich, 1997; e.g., Schommer and Walker, 1995; Hofer, 2000; Buehl et al., 2002; Stahl and Bromme, 2007; for a review see Muis et al., 2006). Beliefs about the nature of knowledge and the nature of knowing represent an important, but not nearly exhaustive set of potential characteristics of a domain.

In sum, we can speculate that between-domain relations of students' academic emotions reflect their judgments of domain characteristics. As the present research aims to test this assumption, knowledge of what actually constitutes an “academic domain” from the perspective of students is highly important. Thus, Study 1 of the series of studies herein reported was to gather characteristics that students attribute to academic domains.

## The role of trait vs. state assessment in between-domain relations of academic emotions

Findings that academic emotions are rather domain specific and that certain domains are more strongly related with respect to students' experience of academic emotions in those domains (e.g., mathematics/physics as compared to mathematics/English) are almost entirely based on reports of habitually experienced academic emotions (trait emotions; e.g., Stipek and Mason, 1987; Marsh and Yeung, 1996; Goetz et al., 2007, 2010a). However, previous research has suggested that reports of habitual emotional experiences can be rather strongly impacted by subjective beliefs (Robinson and Barrett, 2010).

A prominent model that highlights the role of beliefs with respect to the assessment of state vs. trait emotions is Robinson and Clore's (2002) accessibility model of emotional self-report. In this model, state and trait emotional self-reports are differentiated based on the memory system that is activated when reporting feelings. State measures are understood to be *evaluating* individuals' emotions (episodic, experiential), whereas trait measures are thought to reflect individuals' *beliefs* about emotions (semantic, conceptual). In other words, beliefs (e.g., beliefs about academic domain characteristics) are assumed to bias the assessment of trait emotions, whereas reports of real-time emotions in the “here and now” may be less impacted by such beliefs. A number of studies supports this model and shows that it is hardly possible to make conclusions about individuals' actual emotions experienced in real-life situations from their reports of habitual emotions (e.g., Schrader et al., 1990; Buehler and McFarland, 2001; Dewhurst and Marlborough, 2003; Wirtz et al., 2003; Goetz et al., 2013; see also Robinson and Clore, 2002). Drawing upon the accessibility model of emotional self-report, we contended that between-domain relations of *trait* academic emotions would more strongly reflect between-domain relations of domain characteristics, as compared to *state* academic emotions.

## Goals and overview of studies

The present series of three studies are aimed at contributing to our understanding of the structure of academic emotions. The focus of this work was on an intuitive yet largely unexplored area related to the strength of between-domain relations of academic emotions, namely, the characteristics of subject domains. We presumed that the pattern of between-domain relations of academic emotions strongly reflects the similarity of characteristics between different subject domains. Thus, in Study 1 (interview study) our aim was to identify characteristics of academic domains from students' perspectives. The results of this study were a prerequisite for our two subsequent investigations. In Study 2 (questionnaire study) we assessed the identified characteristics and emotional experiences (enjoyment, pride, anxiety, anger, boredom) in different academic domains (mathematics, physics, German, English). We compared patterns of between-domain relations of trait academic emotions with those of domain characteristics and anticipated similar patterns for both constructs. For example, we theorized that mathematics and physics would be judged as being more similar in their characteristics than mathematics and English, and that this would be reflected in between-domain relations of academic emotions. More specifically, we hypothesized that there would be stronger relations between emotions experienced in mathematics and physics as compared to those experienced in mathematics and English. Finally, we examined between-domain relations in real-time academic emotions (Study 3, experience-sampling) and predicted that the pattern of between-domain relations of real-time (state) emotions would show a weaker association with the pattern of between-domain characteristics as compared to trait emotions. This hypothesis was based on the assumption that domain characteristics should be more salient in reports of trait as compared to state emotions. In each of the three studies discussed, two samples of differing age groups were assessed (8th and 11th graders) to provide an indicator of how strongly generalizable results were with respect to students' age. In sum, this research comprises three studies that intend to offer a rather comprehensive picture on how between-domain associations of academic emotions reflect judgments of academic domain characteristics.

### Ethical statement

The procedure was in compliance with the ethical standards (Ethical Principle of the WMA Declaration of Helsinki) and was deemed appropriate by the Institutional Review Board of the University of Konstanz. Participation was voluntary. Written informed consent was obtained from all participants. Furthermore, parents of study participants were informed about the nature of the study and its procedure, and the heads of schools as well as teachers who taught in the classes investigated approved the study protocol. Once the data were collected and entered, all identifiers that could link individual participants to their results were removed and destroyed. Hence, all the analyses were conducted on depersonalized data.

## Study 1—what are the salient characteristics of academic domains from students' perspective?

Different lines of research (e.g., didactics, educational psychology, research on domain knowledge and epistemological beliefs) have tackled the question of what actually constitutes an academic domain. However, each of these lines of inquiry is restricted to a very specific perspective. Further, previous studies that examined students' views on domain characteristics used categories that were predefined by experts (e.g., Jenkins and Nelson, 2005) or investigated how instructors characterized academic domains (e.g., Stodolsky, 1993). Consequently, there is an apparent lack of information on student judgments of salient characteristics of academic domains. The aim of Study 1, therefore, was to glean key characteristics of subject domains as defined by students. Study 1 is a prerequisite to Studies 2 and 3 and a step toward answering our main research question.

### Method

#### Sample and data collection

The study sample included 8th (*N* = 20) and 11th (*N* = 20) graders (each group 50% female) from four different German high schools. The average age of participants was 15.79 years (*SD* = 1.63; grade 8: *M* = 14.24; *SD* = 0.33; grade 11: *M* = 17.34; *SD* = 0.47). Participation was completely voluntary and data collection was anonymous.

#### Measures

Interviews were conducted by trained research personnel and each student interview lasted approximately 10 min. Students were asked the following question, repeated for each of the seven academic domains: “*In your opinion, which properties characterize the school subject [domain]?*” with the order of domains being (1) mathematics, (2) physics, (3) German, (4) English, (5) biology, (6) history, and (7) music. We used single items to keep the length of the interview reasonable as students were asked to answer the question about multiple domains. Students were encouraged to list as many characteristics as they wanted within each domain. If students did not understand the exact wording of the question, or referred to things not related to the question, the interviewer focused the student on characteristics of the subject domains. These probes were intended to help students to focus on the main question without directing their attention to specific characteristics of the domain. Prompts were offered when students deviated into generalities (“Please try to name only the concrete characteristics of the domain”), if students talked about their feelings (“Please try to think only about the concrete characteristics of the domain, not what your feelings are during instruction”), or if students talked about the content of a given subject (“Please try not to talk about the content of the subject, but stick to the concrete characteristics of the domain”). All interviews were audio recorded and transcribed.

#### Data analysis

Qualitative content analysis was used (e.g., Mayring, 2003; Krippendorff, 2007) to build categories of domain characteristics based on students' responses. Loops of inductive category development and deductive category application on randomly selected interviews were used to derive final categories, to which all students' statements could be assigned. Thus, after deleting comments that were unrelated to our questions, and drawing upon specific students' statements, we defined general categories (inductive part) and then categorized these statements according to the specified general categories (deductive part). If a new statement did not fit into existing categories, a new category was added (i.e., a new loop of the inductive-deductive process started). We must note that in order to avoid redundancy in intended analyses of Study 2 and Study 3, we intentionally excluded categories that focused on emotions. Inter-rater reliability based on the final categories was determined by Fleiss' (1971). Kappa, which is a well-established, standardized procedure defined by the ratio of the amount of disproportional concordances to the maximum of attainable concordances:

$$\kappa =\frac{p_{0}-p_{c}}{1-p_{c}}.$$

With *p*_0_ = the proportion of units in which the judges agreed, and *p_c_* = the proportion of units for which agreement is expected by chance (Cohen, 1988). A Kappa-value of κ > 0.60 is considered acceptable (Landis and Koch, 1977). The Kappa-value of the present study was.85, indicating very good inter-rater reliability. Hence, the system of categories affords a rather unambiguous assignment of the students' statements to these categories.

Following the evaluation of student responses, frequency analyses were conducted. If one participant made several statements with identical content, these statements were counted as evidence for only one single category of characteristics. Statements were not emphasized by use of quantifiers; in other words, quantified (e.g., a little, very) and non-quantified statements were valued identically. This procedure resulted in determining the percentage of participants who contributed at least one statement to a specific category. Ambiguous or incoherent statements that did not refer to the question were not included in the results section.

### Results and discussion

Based on qualitative content analyses (Mayring, 2003; Krippendorff, 2007) altogether 13 characteristics of domains were detected. These categories derived from student responses included characteristics of the (1) *quantity of the material* that students are confronted with in a domain, (2) *difficulty of the content* to be learned in a domain, (3) *variety of the content*, the heterogeneity of the content taught in a domain, (4) *coherence of the content*, how strongly different topics in a domain are related to each other, (5) *amount of illustration* in how the material is taught, (6) *relations to everyday life*, to what amount are topics taught in a domain related to students' out of school life (cf., authentic contents), (7) *amount of up-to-date-topics*, how many of the topics taught in the subject refer to topics currently discussed by students and in society, (8) *indisputability of correct task solutions*, the degree to which the correct task solutions are clearly defined, (9) *exchange of views among students*, the degree to which a domain affords students the possibility of exchanging views and thoughts, (10) *wearisomeness*, how physically and mentally tiring and demanding the study is within a subject, (11) *talent necessary for good grades*, how important it is to be talented in a domain in order got get good grades, (12) *value of achievement*, how important it is to get good grades in a domain, and finally (13) *value independent of achievement*, how important a domain is to a student independent of the achievement level reached within this domain. For each of the 13 categories an example of a student statement is presented in Table 1.

**Table 1:** **Study 1—characteristics of domains and sample statements**.

**Characteristics of domains**	**Sample statement**
Quantity of material	German: Really very extensive. Quite a lot of poems. A lot of historical epochs. Really very extensive
Difficulty of content	Physics: My God! Physics is really difficult
Variety of content	You learn and do a lot of different things in German
Coherence of content	In math all new content is always based on the previous one—you can't omit anything
Amount of illustration of material	In physics, unlike math, you can see what happens—the teacher shows you how things work. You see it in experiments
Relations to everyday life	What you have to learn in math has nothing to do with reality and you don't need it in everyday life—except when you aim at becoming a professor of mathematics
Amount of up-to-date topics	In German, as compared to history, you really discuss a lot of current topics—like what you can read in newspapers
Indisputability of correct task solutions	In math you always have one result, which is correct, and everything else is wrong
Exchange of views among students	In English you come to talk to each other, you talk about English poems and other stuff. Sometimes it is quite interesting to hear what others think
Wearisomeness	Math: A lot of calculations and formulas. … It's exhausting
Talent necessary for good grades	Math: You have to be talented—otherwise forget it!
Value of achievement	It is rather important to have good grades in math
Value independent of achievement	English is the most important language of the world. Without English you can't get anywhere—you definitively should be good at it

**Figure 1:**
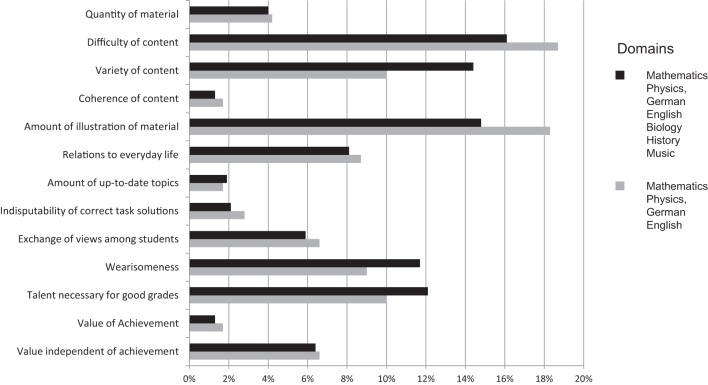
**Frequencies of mentioned characteristics of the domains (multiple responses were possible)**. As compared to the black bars that show the results of the whole assessment (based on 472 statements), the gray bars refer to a subsample of 289 statements related to the four domains which are in the focus of Study 2 and Study 3.

Relative frequencies of all 13 categories are shown in Figure 1. This analysis was based on altogether 472 statements students gave describing characteristics of 7 subject domains (mathematics, physics, German, English, biology, history, music). As the main focus of Studies 2 and 3 concerns the domains of mathematics, physics, German, and English, statements exclusively referring to those domains are also shown in this Figure (based on 289 statements). The Figure shows that the pattern of relative frequencies in the subset of statements referring to only four domains is rather similar to the pattern of relative frequencies referring to seven domains. The three domain characteristics most often mentioned by students were the difficulty and the variety of content, as well as the amount of illustration of the material. Although some of the categories were mentioned rarely by students, they were not eliminated as they seem to be important, even though not often mentioned (e.g., coherence of content, amount of up-to-date topics or indisputability of correct task solutions—a typical epistemological belief).

Thirteen academic domain characteristics were identified based on students' statements gathered in interviews. Although all of the 13 characteristics derived from Study 1 have been mentioned in previous studies (in some cases with different names), the current results revealed categories that were salient to students (as opposed to teachers and professors; e.g., *the degree of subject definition* as outlined by professionals; Stodolsky, 1993). In other words, the 13 categories represent a sub-group of salient characteristics that have been scattered across different lines of research.

## Study 2—do between-domain relations of trait academic emotions reflect between-domain judgments of domain characteristics?

Although studies present convincing evidence that the strength of between-domain reports of habitually experienced, or trait-based, academic emotions differs with respect to particular domain pairs under investigation, it remains unclear why some domains are more strongly related than others^1^. The aim of Study 2 was to examine whether relations of trait academic emotional experiences across certain domains would reflect students' judgments of characteristics related to those domains. We focused on the four domains of mathematics, physics, German, and English and expected to find the strongest relations for the subject pairings of mathematics/physics and German/English.

### Method

#### Sample and data collection

The sample consisted of 855 8th and 854 11th graders (each 49% female) from 74 classes (35/39 in grade 8/11) from 11 different German high schools. The average age of participants was 15.95 years (*SD* = 1.64; grade 8: *M* = 14.42; *SD* = 0.55; grade 11: *M* = 17.51; *SD* = 0.56). Students participated in this study on a voluntary basis, and were tested in their regular classrooms settings. The data were collected by trained testing personnel via a self-report instrument (questionnaire). It took students, on average, 25 min to complete the questionnaire. Eighth-graders have an average of 3/2/4/3 academic hours of mathematics/physics/German/English, and eleventh-graders have an average of 4/3/4/4 academic hours in those domains.

#### Measures

***Characteristics of domains***. A questionnaire was developed based on the 13 characteristics of academic domains found through interviewing students in Study 1. One scale item was formulated for each identified characteristic for the domains of mathematics, physics, German, and English. Reliability of single-items measures cannot be established via conventional methods (e.g., Cronbach's alpha); however, in previous and comparable studies, reliability of single-items was demonstrated, for example, by correlating it with full scales (e.g., meta-analysis by Wanous et al., 1997). Further, a recent study by Gogol et al. (2014) reported findings attesting to high reliability and validity of single-item measures specifically indexing emotional and motivational constructs. Each characteristic within the four domains was presented to students, who answered questions on a 5-point Likert scale (see Table 2). Thus, responses were recorded on altogether 52 questions (13 characteristics × 4 domains). The order of the items was as follows: Characteristic 1 with respect to (a) mathematics, (b) English, (c) physics, (d) German, followed by characteristic 2 with respect to the four domains in the same order up to characteristic 13 (order preserved).

**Table 2:** **Study 2—items on the characteristics of domains—trait**.

**(D)**	**Characteristic of domain**	**Item**	**Response format**	
			**From 1**	**To 5**
D1	Quantity of material	Does the amount of material you have to learn appear negligible, reasonable, or excessive?	Negligible	Excessive
D2	Difficulty of content	Is the difficulty of this subject domain low or high?	Low	High
D3	Variety of content	Is the variety of content in your class low or high?	Low	High
D4	Coherence of content	Are the topics in class incoherent or coherent?	Incoherent	Coherent
D5	Amount of illustration of material	Are there few or many illustrations in this subject domain?	Few	Many
D6	Relations to everyday life	Do the topics of your class relate a little or a lot to everyday life?	Little	A lot
D7	Amount of up-to-date topics	Are there few or many up-to-date topics in this subject domain?	Few	Many
D8	Indisputability of correct task solutions	Are correct task solutions in this subject domain indisputable or disputable?	Indisputable	Disputable
D9	Exchange of views among students	Does this subject domain offer few or many opportunities for exchange of views among students?	Few	Many
D10	Wearisomeness	How wearisome are classes in this subject domain?	A little	A lot
D11	Talent necessary for good grades	Do you need talent to get good grades in this subject domain?	No talent	Talent
D12	Value of achievement	How important is it for you to get good grades in this subject domain?	Unimportant	Important
D13	Value independent of achievement	How important is the subject domain for you independently of the grades?	Unimportant	Important

***Academic emotions***. Two selection criteria were used to identify emotions to be assessed in the current study. First, we aimed to assess emotions that were conceptually distinct on a phenomenological level with respect to a categorization of emotions, based on Watson'S and Tellegen'S (1985) circumplex model. This model uses dimensions of valence and activation to categorize emotions. Second, we searched the research literature for emotions that are particularly salient in academic settings (see Pekrun et al., 2002; Goetz et al., 2007). After combining these two selection criteria, the emotions of enjoyment and pride (positive and activating), anxiety and anger (negative and activating), and boredom (negative and deactivating) were chosen. Positive deactivating emotions (e.g., relief, relaxation, nostalgia) were not used in this study because these emotions tend to occur *after* as opposed to *during* academic situations (see Pekrun et al., 2002). Single-item measures were employed to allow for direct comparison of the results with those of Study 3, in which the use of single survey items is most appropriate (cf., Goetz et al., 2010b; Nett et al., 2011). Reliability of single-item measures has been previously established in similar studies (e.g., Wanous et al., 1997; Nett et al., 2011; for the psychometric properties of single-item measures see Gogol et al., 2014). Questionnaire items were formulated as follows: “*How strongly do you experience [Emotion] in the following subject domains?*” The responses referred to each emotion in the domains of mathematics, physics, German, and English. Response format consisted of a 5-point Likert scale ranging from 1 (*not at all*) to 5 (*very strongly*). The order of the items was as follows: Enjoyment with respect to (a) mathematics, (b) English, (c) physics, (d) German, followed by anger, pride, anxiety, and boredom with respect to the four domains, in the same order.

#### Data analysis

***Calculating similarity indices s(d)s***. The two-level structure of the data with students (Level 1; *N* = 1709) nested within classes (Level 2; *N* = 74) was taken into account when analyzing the data using M*plus* 5.2 software (adopting the “type = complex” option; Muthén and Muthén, 2008). To compare between-domain relations of constructs, we calculated similarity indices *s*(d)s that are based on building mean levels of *z*-standardized correlation coefficients (*z*-standardized due to the fact that correlation coefficients do not represent an interval scale; see Cohen, 1988; for a similar approach see Goetz et al., 2006b). The *s*(d)-values (both with respect to domain characteristics and academic emotions) indicate the strength of between-domain relations for each domain pair and can be interpreted in terms of effect sizes (0.10/0.30/0.50 as small/medium/strong effect; Cohen, 1988).

In a first step, for each domain pair (e.g., mathematics-physics) values of academic characteristics related to the two domains were correlated. As a result, for six domain pairings (based on four academic subjects) 13 (number of characteristics) correlations were calculated, yielding altogether 78 correlations for each grade level. In a second step, all of the correlations were Fisher-*z*-transformed. In a third step, for each of the six domain pairs in each grade level, the arithmetic mean over each of the 13 Fisher-z-transformed correlations referring to the 13 characteristics was calculated. The resulting six (number of domains) mean correlations were re-Fisher-*z*-standardized, resulting in the six *s*(d)-values (mean correlations) for each grade level^2^.

In a similar way, *s*(d)s for academic emotions were calculated. The six *s*(d)-values of academic emotions referring to the domain pairs were based on altogether 30 correlations (5 emotion × 6 domain combinations) for each grade level. Thus, for both characteristics of domains and academic emotions *s*(d)-values show how strongly these constructs are related with respect to two domains (domain pairs). The important feature of the *s*(d)-values is that they can directly be compared across domain pairs (e.g., mathematics-physics vs. mathematics-English) and constructs (domain-characteristics vs. emotions).

***Comparing s(d)s-matrices***. The six *s*(d)-values were presented in a matrix both with respect to domain characteristics (matrix A) and emotions (matrix B). Based on the residual matrix [representing difference scores on the corresponding six *s*(d)-values in matrices A and B] the SRMR (standardized root mean square residual) was calculated to estimate whether the two matrices differ from each other (Bollen, 1989; Hu and Bentler, 1999). The root mean-square residual (RMR) was calculated as

$$\text{RMR}={\left[2\sum_{i\;=\;1}^{p}\sum_{j\;=\;1}^{i}\frac{{\left(s_{ij}-\sigma_{ij}\right)}^{2}}{p\left(p+1\right)}\right]}^{1/2}$$

with *s_ij_* the elements of matrix S (in our case the matrix on domains, matrix A), and σ_*ij*_ the elements of matrix Σ (in this case the matrix on emotions, matrix B); *p* denotes the number of variables, on which the two matrices are based (in this case, *p* = 4 domains, resulting in 4 × 4 matrices). In this expression, *s_ij_* and σ_*ij*_ refer to covariances as represented in the two matrices. However, due to the fact that in the matrices of the present study the *s*(d)-values reflect correlations, the RMR as calculated above reflects the SRMR. According to Hu and Bentler (1998) a SRMR below 0.08 indicates that two matrices do not differ from each other. In this study the SRMR was calculated separately for the 8th- and the 11th-grade sample.

### Results and discussion

For the 8th grade sample, means and standard deviations of the constructs are presented in Table 3. Mean differences (absolute size) across all 13 characteristics as experienced in two different domains (6 combinations of domains) were 0.40 for the mathematics/physics domain pair and 0.31for the German/English domain pair. All values for the other domain pairs were higher ([0.52; 0.60]; median: 0.53). As for emotions, the domains of mathematics and physics revealed rather similar levels, as did the domains of German and English. Mean differences (absolute size) across all six academic emotions as experienced in two different domains (6 combinations of domains) were 0.12 for the mathematics/physics domain pair and 0.16 for the German/English domain pair. All values for the other domain pairs were higher ([0.30; 0.46]; median: 0.39).

**Table 3:** **Study 2—descriptive statistics on constructs—trait—grade 8**.

	**Mathematics**		**Physics**		**German**		**English**	
	***M***	***SD***	***M***	***SD***	***M***	***SD***	***M***	***SD***
**(D) DOMAIN CHARACTERISTICS**								
(D1) Quantity of material	3.51	1.02	3.53	0.97	2.88	1.11	3.14	0.84
(D2) Difficulty of content	3.70	1.54	3.54	1.45	2.94	1.11	3.06	1.13
(D3) Variety of content	2.29	1.36	2.92	1.58	2.97	1.45	3.08	1.38
(D4) Coherence of content	3.48	1.37	3.06	1.17	2.77	1.12	3.30	1.18
(D5) Amount of illustration of material	2.82	1.46	3.80	1.20	2.72	1.07	2.84	1.09
(D6) Relations to everyday life	2.40	1.31	2.55	1.43	3.35	1.56	3.59	1.35
(D7) Amount of up-to-date topics	2.03	1.19	2.60	1.45	3.18	1.40	3.06	1.40
(D8) Indisputability of correct task solutions	3.66	1.88	3.48	1.52	3.01	1.37	3.54	1.08
(D9) Exchange of views among students	2.44	1.50	2.70	1.38	3.46	1.35	3.02	1.29
(D10) Wearisomeness	3.67	1.42	3.40	1.36	3.00	1.12	3.11	1.06
(D11) Talent necessary for good grades	3.10	1.67	2.95	1.59	2.99	1.28	2.52	1.19
(D12) Value of Achievement	4.40	0.76	3.69	1.03	4.10	0.93	4.41	0.65
(D13) Value independent of achievement	3.43	1.55	2.76	1.57	3.40	1.36	4.08	1.14
**(E) ACADEMIC EMOTIONS**								
(E1) Enjoyment	2.44	1.71	2.44	1.69	2.84	1.46	3.08	1.53
(E2) Pride	2.97	1.67	2.84	1.59	3.00	1.26	3.23	1.22
(E3) Anxiety	2.76	2.16	2.59	2.05	2.20	1.53	2.22	1.47
(E4) Anger	3.08	1.85	2.89	1.76	2.58	1.45	2.52	1.43
(E5) Boredom	3.33	1.74	3.46	1.75	3.20	1.55	2.93	1.57

*Response formats: D1, D5, D6, D7, D9, D10 from (1) little to (5) a lot; D2, D3 from (1) low to (5) high; D4 from (1) incoherent to (5) coherent; D8 from (1) indisputable to (5) disputable; D11 from (1) no talent to (5) talent; D12, D13 from (1) unimportant to (5) important. E1-E5 from (1) not at all to (5) very strongly. N = 855*.

As for the 11th grade sample, means and standard deviations of the constructs are outlined in Table 4. Mean differences across characteristics in two different domains were 0.38 for the mathematics/physics domain pair and 0.40 for the German/English domain pair. The values for the other domain pairs were higher [0.84; 0.99]; median: 0.95). As for emotions, the domains of mathematics and physics revealed rather similar levels, as did the domains of German and English. Mean differences across all six academic emotions as experienced in two different domains were.13 for the mathematics/physics domain pair and 0.19 for the German/English domain pair. All values for the other domain pairs were higher ([0.35; 0.59]; median: 0.46).

**Table 4:** **Study 2—descriptive statistics on constructs—trait—grade 11**.

	**Mathematics**		**Physics**		**German**		**English**	
	***M***	***SD***	***M***	***SD***	***M***	***SD***	***M***	***SD***
**(D) DOMAIN CHARACTERISTICS**								
(D1) Quantity of material	3.64	0.98	3.70	0.92	2.56	1.05	2.66	0.84
(D2) Difficulty of content	3.93	1.47	3.81	1.26	2.88	1.24	2.81	1.20
(D3) Variety of content	2.01	1.01	2.50	1.29	2.93	1.37	3.14	1.35
(D4) Coherence of content	3.96	0.98	3.47	1.11	2.70	1.10	2.90	1.16
(D5) Amount of illustration of material	2.93	1.42	3.82	1.12	2.58	1.01	2.69	1.00
(D6) Relations to everyday life	1.96	1.04	2.80	1.39	3.00	1.53	3.75	1.06
(D7) Amount of up-to-date topics	1.58	0.80	2.13	1.22	3.09	1.47	3.70	1.04
(D8) Indisputability of correct task solutions	4.07	1.59	4.05	1.41	2.33	1.32	3.11	1.11
(D9) Exchange of views among students	1.99	1.27	2.21	1.29	3.86	1.24	3.45	1.07
(D10) Wearisomeness	3.76	1.45	3.67	1.25	2.84	1.30	2.76	1.15
(D11) Talent necessary for good grades	3.11	1.53	3.06	1.42	3.47	1.15	2.83	1.19
(D12) Value of Achievement	4.23	0.93	3.75	1.14	3.92	1.09	4.27	0.78
(D13) Value independent of achievement	3.41	1.81	2.84	1.80	3.35	1.59	4.21	0.93
**(E) ACADEMIC EMOTIONS**								
(E1) Enjoyment	2.52	1.70	2.37	1.57	2.86	1.53	3.15	1.39
(E2) Pride	3.09	1.79	2.85	1.66	3.08	1.39	3.24	1.34
(E3) Anxiety	2.95	2.18	2.90	2.09	2.17	1.39	2.16	1.39
(E4) Anger	3.30	1.84	3.19	1.71	2.71	1.41	2.56	1.37
(E5) Boredom	3.21	1.61	3.32	1.66	3.24	1.54	2.92	1.45

*Response formats: D1, D5, D6, D7, D9, D10 from (1) little to (5) a lot; D2, D3 from (1) low to (5) high; D4 from (1) incoherent to (5) coherent; D8 from (1) indisputable to (5) disputable; D11 from (1) no talent to (5) talent; D12, D13 from (1) unimportant to (5) important. E1-E5 from (1) not at all to (5) very strongly. N = 854*.

The results demonstrate that in both samples, students judged domain characteristics of the quantitative domains as being relatively similar. This was also true for the verbal domains. The reported difference between domains was on average stronger in the 11th grade sample as compared to the 8th grade sample. Further, the findings demonstrate that in both the 8th and the 11th grade, students appear to feel similarly within the quantitative domains on the one hand and the verbal domains on the other hand with the difference between domains being slightly stronger in the 11th grade sample as compared to the 8th grade sample.

With respect to the research question, Table 5 shows similarity values *s*(d)s for domain characteristics and academic emotions (see Supplementary material—Appendices A [8th grade sample] and B [11th grade sample] for all 78 correlations for domain characteristics and all 30 correlations for emotions). It is important to note, that all *s*(d)-values as outlined in Table 5 reflect effect sizes and thus can be directly compared. In line with our assumptions, a very similar picture emerged in both samples.

**Table 5:** **Study 2—*s*(d)-values for the trait assessment: between-domain relations of domain characteristics and academic emotions**.

	***s*(d): characteristics of domains**				***s*(d): academic emotions**			
	**M**	**P**	**G**	**E**	**M**	**P**	**G**	**E**
**GRADE 8**								
M	1				1			
P	0.31	1			0.41	1		
G	0.15	0.08	1		0.16	0.19	1	
E	0.17	0.12	0.26	1	0.16	0.15	0.34	
**GRADE 11**								
M	1				1			
P	0.46	1			0.55	1		
G	0.09	0.07	1		0.10	0.09	1	
E	0.10	0.09	0.24	1	0.10	0.10	0.24	1

*s(d): value of the similarity index which reflects mean correlations across all emotions assessed. Mathematics (M), Physics (P), German (G) and English (E). N = 855/854 for grade 8/11*.

More specifically, for the 8th grade sample and domain characteristics, the strongest effects were found for the mathematics/physics domain pair [*s*(d) = 0.31], followed by the German/English domain pair (0.26) and finally, all other domain combinations (0.08 ≤ *s*(d) ≤ 0.15). This pattern parallels the between-domain relations found for academic emotions, in which the strongest effects were also found for mathematics/physics domain pair [*s*(d) = 0.41], followed by the German/English domain pair (0.34) and finally all other domain combinations [0.15 ≤ *s*(d) ≤ 0.19]. In order to statistically test this apparent similarity between the two matrices, we calculated the SRMR. The comparison of matrices revealed the SRMR of 0.05 which is below the cutoff value of 0.08 (Hu and Bentler, 1998) and therefore indicates that the matrices are reasonably similar.

As for the 11th grade sample and domain characteristics, the strongest effects were found for the mathematics/physics [*s(d) = 0.46]* and the German/English (0.24) domain pairs, followed by all other domain combinations [0.07 ≤ *s*(d) ≤ 0.10]. This pattern is similar to the between-domain relations found for academic emotions, in which the strongest effects were also revealed for mathematics/physics [*s*(d) = 0.55], and the German/English (0.24) domain pairs, followed by all other domain combinations [0.09 ≤ *s*(d) ≤ 0.10]. The comparison of matrices revealed the SRMR of 0.03 for which is below the cutoff value of 0.08 (Hu and Bentler, 1998) and therefore indicates that the matrices are reasonably similar.

Results of Study 2 indicated that relations between habitual academic emotional experiences in certain paired domains reflected relations between cognitive judgments of domain characteristics associated with those pairs of domains. Between-domain relations for both types of constructs were, on average, weak to medium, and relations between the domain pairs of mathematics/physics and German/English were relatively strong. Although the present analysis does not allow for a causal interpretation of the results, the strength of between-domain relations of domain characteristics may be interpreted as an antecedent of the strength of relations of reports of habitual academic emotions experienced across those domains. In other words, if students think that certain academic subjects are similar or dissimilar in nature with respect to characteristics of these domains, they might also judge their academic emotional experiences related to those domains as being similar or dissimilar. This causal contingency is supported by appraisal theories of emotion, in which cognitive appraisals, such as judgments of an academic domain, serve first of all as triggers (not consequences) of academic emotions (e.g., Clore, 1994; Roseman, 2001; Roseman and Smith, 2001; Scherer, 2001). However, in considering an alternate causal pathway, reports of habitual emotions may also impact judgments of domain characteristics. For example, a student could attribute feelings of anxiety in a certain subject to a high degree of difficulty that exceeds the student's competence level (see Bandura, 1997; Pekrun, 2006). To establish causal pathways from judgments of domain characteristics to reports of habitual emotions, experimental manipulations of students' cognitive judgments about domains and subsequent examination of the effects of such manipulations on habitual academic emotions are in order.

## Study 3—does the degree of similarity of between-domain relations of academic emotions on the one hand and students' judgments of between-domain characteristics on the other differ with respect to the method of assessing emotions (trait vs. state)?

Reports of real-time, or state, emotional experiences related to an academic domain may be less impacted by subjective beliefs (e.g., perceived coherence of the content students are confronted within a domain) than reports of habitual, or trait, emotions (Robinson and Clore, 2002; Robinson and Barrett, 2010). As judgments of domain characteristics comprise a number of such beliefs, the between-domain relations of trait academic emotions may more strongly reflect judgments of domain characteristics as compared to state academic emotions. To this end, Study 3 aimed to shed light on the role of cognitions in between-domain relations of academic emotions. The same five emotions (enjoyment, pride, anxiety, anger, boredom) across the same four domains (mathematics, physics, German, English) as in Study 2 were examined. In this study, however, the experience sampling method was employed to gauge students' real-time emotional experiences. Whereas Study 2 revealed stronger associations between emotions related to domains that were judged by students as being more similar than others (i.e., mathematics/physics, German/English), we hypothesized in Study 3 that this pattern of relations would be less pronounced in the between-domain relations of state academic emotions.

### Method

#### Sample and data collection

The sample consisted of 58 8th and 63 11th graders (52/49% female in grade 8/11) from 41 classes (21/20 in grade 8/11) from 11 different German high schools. The average age of participants was 15.96 years (*SD* = 1.71; grade 8: *M* = 14.45; *SD* = 0.76; grade 11: *M* = 17.73; *SD* = 0.88). From each of the 41 classes, two to four students were randomly selected to participate in the study. All participants took part in this study on a voluntary basis.

Data were collected using the experience sampling method (Csikszentmihalyi and Larson, 1987; Hektner et al., 2007) over a period of 10 school days. Personal digital assistant (PDA) devices programmed with PMat software (Weiss et al., 2004) were used. In order to obtain representative data of individuals' experiences in four subject domains, the assessment employed a combination of event- and time-randomization procedures (Hektner et al., 2007). Students were instructed to activate their device at the beginning of every mathematics, physics, German, and English classes. The PDA device would signal the participant to answer a digital questionnaire at a randomly chosen moment within the 45 min lesson. All teachers were informed of the experimental procedure at the beginning of the study and they gave their agreement to proceed, as did the students. Each student completed up to 28 experience sampling questionnaires. Students completed on average 12.61 questionnaires throughout the 10 school days (*SD* = 6.04; minimum = 1, maximum = 28). On average, 3.60, 2.50, 3.35, and 3.16 of the questionnaires were related to mathematics, physics, German, and English, respectively.

#### Measures

To avoid overly intrusive questionnaires, state constructs were assessed using single-item measures. This practice is consistent with findings from Wanous et al. (1997) indicating that single-item measures of job satisfaction correlate highly with multi-item scales. Previous experience sampling studies on academic emotions also support the viability of this approach (Goetz et al., 2010b, 2014; Nett et al., 2011). Finally, a recent analysis by Gogol et al. (2014) revealed high reliability and validity of single-item measures on emotional and motivational constructs. The intensity of the emotions of enjoyment, pride, anger, anxiety, and boredom were assessed by the items “*How much* [EMOTION] *are you experiencing during this class?*” (for a similar assessment see Goetz et al., 2012a). Response format for these items consisted of a five-point Likert scale ranging from 1 (*not at all*) to 5 (*very strongly*).

#### Data analysis

To analyze the strength of between-domain relations of state academic emotions, similarity indices *s*(d)s were calculated by employing the procedure delineated in Study 2 (cf., Cohen, 1988), using the same five emotions (enjoyment, pride, anxiety, anger, boredom) crossed with each of the same four domains (mathematics, physics, German, English). As in Study 2, the *s*(d)-values indicate the strength of between-domain relations of academic emotions for each domain pair, and can be interpreted in terms of effect sizes (0.10/0.30/0.50 as small/medium/strong effect; Cohen, 1988). Also, similarly to Study 2 the matrix containing the *s*(d)s-values for state emotions was compared with the matrix containing the *s*(d)s-values for domain characteristics (derived in Study 2).

### Results and discussion

Means and standard deviations of constructs are outlined in Table 6. For both sub-samples, the levels of academic emotions were rather similar across the four academic domains. For the 8th grade sample the range of mean values of the differences (absolute size) across all six academic emotions as experienced in two different domains (6 combinations of domains, that is 6 values for each grade level) was [0.06; 0.27] and [0.05; 0.19] for the 11th grade sample. The median of those values was 0.17 for the 8th grade sample and 0.15 for the 11th grade sample, showing that students' reported emotional experiences were very similar across the four academic domains.

**Table 6:** **Study 3—state assessment: descriptive statistics on constructs**.

**Academic emotions**	**Mathematics**		**Physics**		**German**		**English**	
	***M***	***SD***	***M***	***SD***	***M***	***SD***	***M***	***SD***
**GRADE 8**								
Enjoyment	2.15	0.67	2.21	0.67	2.37	0.95	2.77	0.95
Pride	1.63	0.55	1.65	0.61	1.55	0.44	1.77	0.61
Anxiety	1.56	0.40	1.58	0.51	1.34	0.37	1.38	0.35
Anger	1.80	0.76	1.82	0.79	1.59	0.48	1.55	0.48
Boredom	3.10	1.10	2.94	1.36	2.97	1.00	3.14	1.07
**GRADE 11**								
Enjoyment	2.23	0.92	2.24	1.03	2.36	1.09	2.46	1.19
Pride	1.71	0.76	1.64	0.71	1.59	0.63	1.96	1.09
Anxiety	1.44	0.54	1.32	0.47	1.28	0.41	1.30	0.27
Anger	1.79	1.03	1.83	0.85	1.81	1.23	1.75	0.73
Boredom	2.76	1.27	3.24	1.57	3.20	1.46	3.09	1.03

*Response formats: (1) not at all to (5) very strongly. N = 58/63 for grade 8/11 at student level*.

With respect to the research question, Table 7 shows the between-domain correlations of real-life academic emotions (see Supplementary material—Appendix C for all 30 correlations for the 8th and 11th graders). In line with our assumptions, *s*(d)-values (similarity indices) were rather consistent for both age groups ([0.21; 0.31] in grade 8; [0.10; 0.22] in grade 11). The pattern of relations did not consistently show stronger between-domain associations for the mathematics/physics and German/English domain pairs as compared to other possible domain combinations. In terms of effect sizes [*s*(d)-values], all relations were weak to medium.

**Table 7:** **Study 3—s(d)-values for the state assessment: between-domain relations of academic emotions**.

	***s*(d): academic emotions**			
	**M**	**P**	**G**	**E**
**GRADE 8**				
M	1			
P	0.27	1		
G	0.26	0.31	1	
E	0.24	0.21	0.28	
**GRADE 11**				
M	1			
P	0.22	1		
G	0.15	0.10	1	
E	0.12	0.11	0.11	1

*s(d): value of the similarity index that reflects mean correlations across all emotions assessed. Mathematics (M), Physics (P), German (G) and English (E). N = 58/63 for grade 8/11 at student level*.

When comparing all *s*(d)-values for state academic emotions with those of domain characteristics (from Study 2), the revealed SRMR was 0.09 for both the 8th and the 11th grade. These values were clearly higher than those found in Study 2 (trait emotions) indicating that the matrices were reasonably different from each other. Even more important is the fact that the differences were stronger in Study 3 as compared to Study 2. In sum, the between-domain relations of trait academic emotions more strongly reflect interrelations of domains with respect to students' judgments of domain characteristics, as compared to assessment of state, or real-time, academic emotional experiences.

Although the trait assessment revealed that the domain pairs of mathematics/physics and German/English were judged as being more similar than other pairs (both with respect to domain characteristics and academic emotions), this pattern was not found in the state assessment of academic emotions. For example, in terms of effect sizes, the differences in similarity indices *s*(d) related to mathematics/physics and *s*(d) related to mathematics/German were 0.16/0.37 for the trait assessment and 01/0.07 for the state assessment for grade levels 8/11. Although differences in *s*(d) in the trait assessment strongly reflected the difference in *s*(d) related to the judgments of domains (0.25/0.45 for grade levels 8/11), the differences in *s*(d) in the state assessment did not.

It is important to note that the data of Study 2 and 3 were not assessed with the same sample. However, both studies included two sub-samples (8th and 11th graders) and the results across age groups were very similar, all of which provides strong support of study hypotheses.

## General discussion

The aim of the present research was to contribute to the existing knowledge regarding the pattern of between-domain relations of students' academic emotions. Previous research has shown that between-domain relations of trait academic emotions strongly differ with respect to the domain pairs under investigation. The focus of the present study was to contribute to our understanding of why such patterns of between-domain relations of trait academic emotions arise.

### Students' perspectives on salient domain characteristics

One of the main goals of the current study was to investigate whether the pattern of between-domain relations in trait academic emotions reflected similarities and dissimilarities of academic subject domains as perceived by students. To accomplish this goal, students' perspectives on the most salient characteristics of academic domains were gathered (Study 1). Thirteen main characteristics were identified from student interviews, including the difficulty of content, the value of achievement, and the amount of illustration of the material. From a practical perspective, these categories may assist educators in understanding what aspects students focus on when thinking about subject domains.

### Between domain relations of academic emotions and students' thoughts about domain characteristics

To further address the main aims of this research, thirteen characteristics identified in Study 1 were assessed in combination with the assessment of five trait academic emotions (enjoyment, pride, anxiety, anger, boredom) in the four subject domains of mathematics, physics, German, and English (Study 2, questionnaire study, trait). Consistently with the study hypothesis, rather similar patterns for between-domain relations of domain characteristics on the one side and academic domains on the other side were revealed. According to the assumptions that cognitions about domains are first of all salient in trait assessments, we presumed that similar patterns for between-domain relations of domain characteristics and academic emotions would be detected in trait but not in state assessments of academic emotions. To this end, between-domain relations of real-time emotions were examined (Study 3, experience-sampling, state).

In line with this assumption, Study 3 indicated that the pattern of between-domain relations of state emotions did not reflect the pattern of between-domain relations of domain characteristics. In sum, the two main studies herein reported (Studies 2, 3) showed that the between-domain structure of academic emotions (trait) reflects the structure of cognitive domain judgments, whereas real-time emotions (state) do not, thus supporting previous research.

The results indicate that if students report that they feel more similarly in some domains vs. others, it may be the result of thinking that the respective domains are more similar in nature—without really *feeling* more similar in those domains. This supports previous reports in the literature showing that trait emotions more strongly reflect what we *think about our feeling*, and not just what we *really feel* (Robinson and Clore, 2002). It is important to take this conceptual difference into account when interpreting trait reports on academic emotional experiences. For example, Goetz et al. (2012a) demonstrated that girls reported higher levels of trait mathematics anxiety as compared to boys, whereas boys and girls showed similar levels of state mathematics anxiety. Further, they showed that this discrepancy in trait and state reports could partly be explained by females' lower mathematics-related academic self-concept (although having similar grades in mathematics). The study by Goetz et al. (2012a) also showed that cognitions played a crucial role for the discrepancy of trait vs. state emotions. Our study contributes to the field of “thinking what we feel” vs. “really feeling” and our findings may serve as a stepping stone for future studies examining the role of thinking about domains in trait and state emotional self-reports.

### Summary of results and conclusion

The studies herein reported demonstrated that the degree of similarity between the pattern of between-domain relations of domain characteristics and the pattern of between-domain relations of academic emotions differs with respect to the method of assessment (trait vs. state). It is important to note that *both* state and trait reports on academic emotions are important. State emotions show what people “really” feel and consequently, they are highly important with respect to psychological and physiological health. As for trait emotions, they are highly important for decision-making processes (cf., Wirtz et al., 2003). For example, in the context of career decisions (e.g., choosing to go into a math intensive field) *what we think we feel* in specific domains (e.g., mathematics) may be more important than *what we really feel*. Thus, both trait and state emotions matter, and it might be misleading to suggest that one approach would be “better” than the other. It is advisable, however, that researchers and practitioners alike bear in mind the fact that state and trait reports on academic emotions represent different conceptual entities.

## Limitations and practical implications

There are some limitations as well as a number of practical implications related to our studies. One limitation is that in Study 1 only one interview question was used for eliciting students' responses about domain characteristics. Because we targeted seven different academic domains, it was not feasible to ask additional questions due to potential weariness resulting from the repetitive nature of such interview. Future studies might focus on fewer domains and assess salient domain characteristics from students' perspective in more detail. Another potential shortcoming of the present series of studies is that the employed design did not allow for establishing a causal ordering of effects. Although theoretical approaches (e.g., appraisal-based emotion theories; e.g., Pekrun, 2006) suggest that beliefs represent the key antecedent of emotions, emotions might in turn impact belief systems (i.e., long-term). Further, only four academic domains were included in Studies 2 and 3. Concerning the sample, the two sub-samples were restricted to adolescents, namely 8th and 11th graders. Characteristics of domains were investigated in Study 2 (questionnaire study) but not in Study 3 (experience sampling). As for the assessment in Study 3, reports of state emotions may have been influenced by factors that were not directly related to the domains (e.g., emotions resulting from thoughts related to future events). Although such effects should be random across assessments and students, they might have contributed to the pattern of correlations among state emotions and consequently, to the degree to which this pattern differs from those of characteristics of domains. Future studies might broaden our understanding of between-domain relations of academic emotions by taking the shortcomings of the present research into account.

The results of the present studies might be important for practitioners with respect to judging students' emotions in academic domains. Teachers tend to view students' individual characteristics as habitual, domain-general attributes, rather than as domain-specific phenomena (Marsh et al., 1983; Marsh, 1993; Pohlmann et al., 2004). The findings of the present research suggest that it may be more misleading than previously assumed to generalize students' academic emotions from one domain to another.

Further, it is important for teachers to know that levels of habitual emotions reported by a student with respect to a specific domain (e.g., “I generally experience high levels of anxiety in mathematics”) might differ from what he or she *really* feels in this domain. Both levels are important in the educational context. For example, habitual emotions are critical in regards to career intentions (cf., Wirtz et al., 2003), whereas state emotions matter for classroom behavior. The results of the present research are in support of the assumption that reports of habitual emotions might be impacted by judgments of characteristics of domains. For example, judging a domain as being rather difficult might result in students' overestimation of the level of anxiety experienced in this domain.

This is in line with a recent finding showing that compared to boys, girls overestimate their math anxiety due to their lower math-related academic self-concept despite having similar grades as boys (Goetz et al., 2013). Consequently, when judging students' academic emotions it is important to listen to their reports but also to focus on specific and often subtle indicators of emotions based on students' behavior in class (see Frenzel and Stephens, 2013 for a detailed description of useful indicators and tools for assessing student emotions in the classroom). Consequently, the present findings may contribute to optimize teachers' behavior in the classroom and develop sensitivity to students' emotions.

### Conflict of interest statement

The authors declare that the research was conducted in the absence of any commercial or financial relationships that could be construed as a potential conflict of interest.

## Footnotes

^1^ It is important to note that this study refers to a traditional definition of trait emotions, in which traits are seen as “typical” emotional experiences that occur consistently in specific contexts (e.g., math domain; Cattell and Scheier, 1961; Spielberger, 1972), which are in the present study academic domains. Thus, in our study trait academic emotions reflect “typical” emotions experienced within but not across domains.

^2^ We would like to note that we used this approach [i.e., derived s(d)-values based on 13 single correlations for each domain pair] over some seemingly viable alternatives. For example, we chose against building factors of characteristics because, per our research goals and data structure, each characteristic should have been weighted the same way. So, the results of multi-level factor analyses showed that factor loadings of domain characteristics strongly differed across academic domains. It is further important to note that high correlations among single domain characteristics (i.e., representing one underlying factor) are not required for this kind of analyses. Judgments about the degree of similarity among different domains can be based on partly unrelated dimensions.
