# Supplementary material for: Between-domain relations of students' academic emotions and their judgments of school domain similarity
Source: Front Psychol. 2014 Oct 21;5:1153. doi: 10.3389/fpsyg.2014.01153 (PMC4204457; doi:10.3389/fpsyg.2014.01153)
Supplement: Supplementary file 1 [file Table1.PDF]

Appendix

A *Study 2 - Trait*: *Between-Domain Relations of Domain Characteristics and Academic Emotions – grade 8*

	M	P	G	E	M	P	G	E	M	P	G	E	M	P	G	E
	(D1) Quantity of Material				(D2) Difficulty of Content				(D3) Variety of Content				(D4) Coherence of content.			
M	1				1				1				1			
P	.21**	1			.40**	1			.26**	1			.22**	1		
G	.15**	–.01	1		.04	–.01	1		.08*	.08	1		.12**	.22**	1	
E	.04	.07	.13*	1	.07	.03	.25**	1	.09	.09*	.25**	1	.12**	.13**	.19**	1
	(D5) Amount of illustr. of mat.				(D6) Relations to Everyd. Life				(D7) Amount of up-to-date topics				(D8) Indisput. of correct task sol			
M	1				1				1				1			
P	.28**	1			.26**	1			.39**	1			.46**	1		
G	.15**	.08*	1		.19**	.05	1		.07*	.07	1		–.23**	–.20**	1	
E	.11**	.17**	.23**	1	.17**	.16**	.29**	1	.26**	.29**	.15**	1	–.06	.02	.23**	1
	(D9) Exch. of views among stud.				(D10) Wearisomeness.				(D11) Talent nec. for good grades				(D12) Value of Achievement			
M	1				1				1				1			
P	.31**	1			.28**	1			.28**	1			.38**	1		
G	.00	.07	1		.13**	–.05	1		.15**	.08*	1		.44**	.15**	1	
E	.27**	.24**	.19**	1	.16**	.04	.22**	1	.11**	.17**	.23**	1	.44**	.25**	.52**	1
	(D13) Value indep. of achievem				(E1) Enjoyment				(E2) Pride				(E3) Anxiety.			
M	1				1				1				1			
P	.46**	1			.35**	1			.49**	1			.51**	1		
G	.20**	.05	1		.03	.10**	1		.18**	.19**	1		.25**	.30**	1	
E	.26**	.11**	.34**	1	.06	.00	.27**	1	.20**	.19**	.37**	1	.27**	.21**	.41**	1
					(E4) Anger				(E5) Boredom							
M					1				1							
P					.42**	1			.28**	1						
G					.20**	.20**	1		.15**	.16**	1					
E					.14**	.18**	.37**	1	.13**	.15**	.30**	1				

Note. Mathematics (M), Physics (P), German (G) and English (E). D1 to D13: Domain Characteristics; E1 to E5: Emotions. $*p < .01$; $**p < .001$; $N = 855$.

B *Study 2 - Trait*: *Between-Domain Relations of Domain Characteristics and Academic Emotions – grade 11*

(D1) Quantity of Material

	M	P	G	E
M	1			
P	.45**	1		
G	.05	.00	1	
E	.08*	.06	.24**	1

(D2) Difficulty of Content

	M	P	G	E
M	1			
P	.58**	1		
G	–.07*	–.07	1	
E	.02	.07	.29**	1

(D3) Variety of Content

	M	P	G	E
M	1			
P	.31**	1		
G	–.02	.01	1	
E	.05	.04	.12	1

(D4) Coherence of content.

	M	P	G	E
M	1			
P	.29**	1		
G	–.05	.08	1	
E	–.05	.05	.20**	1

(D5) Amount of illustr. of mat.

	M	P	G	E
M	1			
P	.23**	1		
G	.07	–.08*	1	
E	–.04	–.06	.16**	1

(D6) Relations to Everyday Life

	M	P	G	E
M	1			
P	.34**	1		
G	.01	–.01	1	
E	.08*	.13**	.25**	1

(D7) Amount of up-to-date topics

	M	P	G	E
M	1			
P	.46**	1		
G	.03	.03	1	
E	.05	.06	.20**	1

(D8) Indisput. of correct task sol.

	M	P	G	E
M	1			
P	.64**	1		
G	–.35**	–.30**	1	
E	–.21**	–.15**	.35**	1

(D9) Exch. of views among stud.

	M	P	G	E
M	1			
P	.51**	1		
G	–.07	–.06	1	
E	.07	.02	.29**	1

(D10) Wearisomeness.

	M	P	G	E
M	1			
P	.49**	1		
G	–.06	.00	1	
E	.03	.06	.11*	1

(D11) Talent nec. for good grades

	M	P	G	E
M	1			
P	.54**	1		
G	.07	.02	1	
E	.17**	.19**	.21**	1

(D12) Value of Achievement

	M	P	G	E
M	1			
P	.53**	1		
G	.32**	.13**	1	
E	.30**	.18**	.46**	1

(D13) Value indep. of achievem

	M	P	G	E
M	1			
P	.52**	1		
G	.02	–.09*	1	
E	.12**	.04	.26**	1

(E1) Enjoyment

	M	P	G	E
M	1			
P	.48**	1		
G	–.08*	–.06	1	
E	–.06	–.06	.17**	1

(E2) Pride

	M	P	G	E
M	1			
P	.61**	1		
G	.16**	.09	1	
E	.15**	.14**	.34**	1

(E3) Anxiety.

	M	P	G	E
M	1			
P	.71**	1		
G	.22**	.26**	1	
E	.23**	.27**	.43**	1

(E4) Anger

	M	P	G	E
M	1			
P	.50**	1		
G	–.03	–.02	1	
E	–.02	–.02	.13*	1

(E5) Boredom

	M	P	G	E
M	1			
P	.38**	1		
G	–.02	–.04	1	
E	.03	–.03	.12*	1

Note. Mathematics (M), Physics (P), German (G) and English (E). D1 to D13: Domain Characteristics; E1 to E5: Emotions. *$p < .01$; **$p < .001$; $N = 854$

C *Study 3 – State-Assessment: Between-Domain Relations of Academic Emotions*

	M	P	G	E	M	P	G	E	M	P	G	E
**Grade 8**												
	Enjoyment				Pride				Anxiety			
M	1				1				1			
P	.32	1			.23	1			.28	1		
G	.21	.39**	1		.41**	.45**	1		.28*	.04	1	
E	.23*	.12	.18	1	.38**	.17	.42**	1	.33*	.16	.31*	1
	Anger				Boredom							
M	1				1							
P	.28	1			.22	1						
G	.09	.20	1		.28	.44**	1					
E	.10	.26	.26	1	.16	.33*	.19	1				
**Grade 11**												
	Enjoyment				Pride				Anxiety			
M	1				1				1			
P	.12	1			.45**	1			–.09	1		
G	.22	–.12	1		.17	.08	1		.09	–.06	1	
E	–.02	.21*	.10	1	.27	–.06	–.07	1	.04	.03	.04	1
	Anger				Boredom							
M	1				1							
P	–.09	1			.27	1						
G	.09	–.06	1		–.12	.02	1					
E	.04	.03	.04	1	.08	.12	.12	1				

Note. Mathematics (M), Physics (P), German (G) and English (E). *p < .01; **p < .001; *N* = 58/63 for grade 8/11 at student level.
